# DNA polymerase switching: effects on spontaneous mutagenesis in *Escherichia coli*

**DOI:** 10.1111/j.1365-2958.2008.06526.x

**Published:** 2008-11-20

**Authors:** Elena Curti, John P McDonald, Samantha Mead, Roger Woodgate

**Affiliations:** Laboratory of Genomic Integrity, National Institute of Child Health and Human Development, National Institutes of HealthBethesda, MD 20892-3371, USA

## Abstract

*Escherichia coli* possesses five known DNA polymerases (pols). Pol III holoenzyme is the cell's main replicase, while pol I is responsible for the maturation of Okazaki fragments and filling gaps generated during nucleotide excision repair. Pols II, IV and V are significantly upregulated as part of the cell's global SOS response to DNA damage and under these conditions, may alter the fidelity of DNA replication by potentially interfering with the ability of pols I and III to complete their cellular functions. To test this hypothesis, we determined the spectrum of *rpoB* mutations arising in an isogenic set of *mutL* strains differentially expressing the chromosomally encoded pols. Interestingly, mutagenic hot spots in *rpoB* were identified that are susceptible to the actions of pols I–V. For example, in a *recA730 lexA*(Def) *mutL* background most transversions were dependent upon pols IV and V. In contrast, transitions were largely dependent upon pol I and to a lesser extent, pol III. Furthermore, the extent of pol I-dependent mutagenesis at one particular site was modulated by pols II and IV. Our observations suggest that there is considerable interplay among all five *E. coli* polymerases that either reduces or enhances the mutagenic load on the *E. coli* chromosome.

## Introduction

Cellular survival relies upon the ability of an organism to faithfully duplicate its genome. While an occasional mutation may prove to be a selective advantage in evolutionary terms, most mutations are deleterious. Indeed, replication errors in humans often lead to genetically inheritable diseases, the onset of cancer and premature ageing (Kunkel, 2003; McCulloch and Kunkel, 2008). As a consequence, how a cell chooses a particular DNA polymerase (pol) to maintain its genomic integrity is of great interest. In humans, it is particularly challenging, as the genome is now believed to encode at least 15 DNA polymerases (Bebenek and Kunkel, 2002). As might be expected, lower life forms tend to possess fewer polymerases, but as in the case of *Escherichia coli*, there are still five to choose from, which is nevertheless a daunting task.

*Escherichia coli* pol I was the first DNA polymerase ever identified (Bessman *et al*., 1956). It is encoded by the *polA* gene (De Lucia and Cairns, 1969) and is estimated to be present at an intracellular concentration of ∼400 molecules per cell (Kornberg and Baker, 1992). The single 103 kDa pol I polypeptide not only exhibits polymerase activity, but 5′→3′ and 3′→5′ exonuclease activities (Kornberg and Baker, 1992). Pol I's primary role is to remove RNA primers and fill Okazaki fragments generated during lagging strand DNA synthesis (Okazaki *et al*., 1971), but it also fills gaps generated during nucleotide excision repair of damaged DNA (Cooper and Hanawalt, 1972; Kornberg and Baker, 1992; Friedberg *et al*., 2006). Δ*polA* strains are inviable when grown in rich medium, but are viable if the 5′→3′ or 3′→5′ exonuclease functions are provided *in trans* (Joyce and Grindley, 1984), indicating that exonuclease and not polymerase functions are primarily required for viability.

Pol II activity was first described in 1971 (Kornberg and Gefter, 1971; Gefter *et al*., 1971), but it was not until 1990 that the *polB* gene encoding pol II was shown to be allelic with the LexA-regulated and damage-inducible *dinA* gene (Bonner *et al*., 1990; Iwasaki *et al*., 1990). In an uninduced cell, pol II is expressed at a basal level of ∼50 molecules per cell (Qiu and Goodman, 1997) and these levels increase approximately sevenfold upon DNA damage (Bonner *et al*., 1988). The single 88 kDa pol II polypeptide also exhibits for both polymerase and 3′→5′ exonuclease activities (Cai *et al*., 1995) and as a consequence, pol II-dependent replication is reasonably accurate with misincorporations occurring in the 1 × 10^−6^ range (Cai *et al*., 1995). For many years, the cellular role of pol II remained enigmatic, but it is now known that the polymerase plays an important role in replication restart (Rangarajan *et al*., 1999; 2002) and translesion replication (Napolitano *et al*., 2000; Becherel and Fuchs, 2001). It has also been suggested that pol II's proofreading activity may also help maintain *E. coli*'s genomic integrity by removing replication errors generated by pol III (Banach-Orlowska *et al*., 2005).

Pol III holoenzyme is the cell's main replicase (Gefter *et al*., 1971; Wechsler *et al*., 1973; McHenry and Kornberg, 1977; Kornberg and Baker, 1992). The holoenzyme is encoded by 10 separate genes, which comprise a 15-polypeptide replicase (McHenry, 1991; 2003; O'Donnell, 2006; Pomerantz and O'Donnell, 2007). Pol III holoenzyme is highly processive and the estimated 10–20 molecules per cell are sufficient to facilitate duplication of the entire ∼4 Mbp *E. coli* genome. The 130 kDa α-catalytic subunit is encoded by *dnaE* (Gefter *et al*., 1971) and temperature-sensitive missense mutations in *dnaE* often exhibit elevated spontaneous mutation rates, even at permissive temperature (Hall and Brammar, 1973; Wechsler *et al*., 1973; Sevastopoulos and Glaser, 1977; Konrad, 1978).

Pol IV activity was first reported in 1999 and shown to be encoded by the damage-inducible *dinB* gene (Wagner *et al*., 1999). However, the LexA-binding site within the *dinB* operator varies considerably from the consensus binding site (Fernandez de Henestrosa *et al*., 2000). As a consequence, pol IV is expressed at high basal levels, with an estimated intracellular concentration of ∼250 molecules per undamaged cell and these levels increase a further 10-fold upon DNA damage (Kim *et al*., 2001). The enzyme lacks intrinsic 3′→5′ exonuclease activity and is a low-fidelity enzyme with a misincorporation frequency in the 10^−3^−10^−4^ range (Tang *et al*., 2000). While it is believed that basal levels of error-prone pol IV do not contribute significantly to spontaneous mutagenesis in dividing cells (Wolff *et al*., 2004; Kuban *et al*., 2005; Tago *et al*., 2005), stationary phase cells exhibit *dinB*/pol IV-dependent mutagenesis (McKenzie *et al*., 2001; Tompkins *et al*., 2003) and overproduction of pol IV increases −1 frameshift mutagenesis in exponentially growing cells (Kim *et al*., 1997; Wagner and Nohmi, 2000; Kuban *et al*., 2005). In addition, it has been recently suggested that pol IV may also contribute to SOS-dependent spontaneous base-pair substitution mutagenesis (Kuban *et al*., 2006).

Pol V was the last *E. coli* polymerase identified (Tang *et al*., 1999). The enzyme is encoded by *umuDC* (Tang *et al*., 1999), and consists of a dimer of the post-translationally modified UmuD′ protein in a complex with UmuC (Woodgate *et al*., 1989; Reuven *et al*., 1999; Tang *et al*., 1999). Like *dinA*/*polB* and *dinB*/(pol IV) the *umuDC* genes are upregulated in response to DNA damage (Bagg *et al*., 1981). However, unlike pol II and pol IV, pol V is expressed at very low basal levels. Indeed, LexA-mediated transcriptional control, combined with RecA-mediated intermolecular auto-cleavage of UmuD (McDonald *et al*., 1998) required to activate UmuD′ (Nohmi *et al*., 1988), as well as Lon- and ClpXP-mediated proteolysis of the Umu proteins (Frank *et al*., 1996; Gonzalez *et al*., 1998; 2000), limits the number of functionally active pol V molecules in an undamaged cell to 15 or less (Woodgate and Ennis, 1991). Levels of pol V do, however, increase upon DNA damage, but at most reach a maximum of 200 molecules per wild-type cell, which is roughly equivalent to basal levels of pol IV expression (Woodgate and Ennis, 1991; Kim *et al*., 2001). Like pol IV, pol V lacks intrinsic 3′→5′ proofreading activity and is also considered a low-fidelity polymerase (Tang *et al*., 2000). Despite its low cellular concentration, pol V is responsible for most damage-induced mutagenesis in *E. coli*, as strains carrying certain missense mutations in *umuD* or *umuC* (Kato and Shinoura, 1977; Steinborn, 1978) or a deletion of the entire *umuDC* operon (Woodgate, 1992) are essentially rendered non-mutable, even after exposure to a wide variety of known mutagens/carcinogens.

All five *E. coli* pols bind the replicative β-clamp and this interaction is required for both processive synthesis *in vitro* and the respective enzyme's cellular functions (Bonner *et al*., 1992; Dalrymple *et al*., 2001; Lopez de Saro and O'Donnell, 2001; Pham *et al*., 2001; Wagner *et al*., 2001; Becherel *et al*., 2002; Lenne-Samuel *et al*., 2002; Bunting *et al*., 2003). While pol I (*polA*) and pol III (*dnaE*) are essential, strains carrying deletions of pol II (*polB*), pol IV (*dinB*) or pol V (*umuDC*) are viable, but strains lacking one or more of the pols are unable to compete with polymerase-proficient bacteria during the stationary phase of the bacterial life cycle, indicating that the three damage-inducible polymerases each contribute to the overall selective fitness of *E. coli* (Yeiser *et al*., 2002).

Given its high fidelity and processivity, it is generally assumed that pol III facilitates duplication of the *E. coli* chromosome with the assistance of pol I to fill Okazaki fragments (Kornberg and Baker, 1992). However, recent studies with replication-impaired pol III mutants suggest that pols II, IV and V may have considerable access to genomic DNA (Strauss *et al*., 2000; Vandewiele *et al*., 2002; Sutton, 2004; Gawel *et al*., 2008). As noted above, the cellular concentrations of *E. coli*'s five polymerases varies considerably and we were interested in investigating the possibility that under certain conditions, pols II, IV or V might compete with wild-type pols I and III, so as to influence the overall fidelity and/or spectrum of mutations arising on the *E. coli* chromosome. To do so, we analysed the spectrum of missense mutations generated in *rpoB* in an isogenic set of *mutL* strains differentially expressing one or more of *E. coli*'s five pols. Our study was facilitated by the fact that each polymerase leaves a unique genetic ‘fingerprint’ (Wolff *et al*., 2004) and that the strains utilized lack methyl-directed mismatch repair, so that we could follow polymerase-specific misincorporation events, rather than assay those events simply remaining after repair (Schaaper and Dunn, 1987). Interestingly, in the various polymerase-deficient backgrounds assayed, the location and magnitude of the base substitution hot spots changed, suggesting that under certain conditions, there is considerable interplay between *E. coli*'s pols that ultimately either reduces or enhances the mutagenic load on the *E. coli* genome.

## Results

### Mutation rates in the various MutL strains

The strains used in this study harbour Tn*5* or Tn*10* insertions in *mutL* and are deficient in postreplicative methyl-directed mismatch repair (Table 1). As a consequence, they exhibit mutations rates that are ∼100–200-fold higher than a mismatch-proficient strain (Table 2). In general, the mutation rates of the various *mutL* strains were similar and only varied from the parental *recA*^+^*lexA*^+^*mutL* strain, RW720, by a factor of two- to fourfold (Table 2). The spectra of *rpoB* mutations were obtained from ∼350–400 individual *rpoB* mutants arising in each strain background.

**Table 1:** *E. coli* strains used in this work.

Strain	Relevant genotype
RW118^a^	*recA*^+^*lexA*^+^*mutL*^+^
RW720^a^	*recA*^+^*lexA*^+^*mutL211*::Tn*5*
RW740^a^	*recA*^+^*lexA*^+^*dnaE486 zae502*::Tn*10 mutL211*::Tn*5*
RW766^a^	*recA*^+^*lexA*^+^*mutL218*::Tn*10*/pCJ102^c^
RW742^a^	*recA*^+^*lexA*^+^Δ*polA*::kan *mutL218*::Tn*10/*pCJ102^c^
RW722^a^	*recA*^+^*lexA51*(Def) *mutL211*::Tn*5*
RW690^a^	*recA730 lexA51*(Def) *mutL211*::Tn*5*
RW694^a^	*recA730 lexA51*(Def) Δ(*umuDC*)*596*::*ermGT mutL211*::Tn*5*
RW604^b^	*recA730 lexA51*(Def) *srlC300*::Tn*10*Δ*umuDC595*::*cat mutL211*::Tn*5*
RW708^a^	*recA730 lexA51*(Def) Δ*dinB61*::*ble mutL211*::Tn*5*
RW712^a^	*recA730 lexA51*(Def) Δ*polB*::ΩSpc *mutL211*::Tn*5*
RW710^a^	*recA730 lexA51*(Def) Δ(*umuDC*)*596*::*ermGT*Δ*dinB61*::*ble mutL211*::Tn*5*
RW714^a^	*recA730 lexA51*(Def) Δ(*umuDC*)*596*::*ermGT*Δ*polB*::ΩSpc *mutL211*::Tn*5*
RW716^a^	*recA730 lexA51*(Def) Δ*dinB61*::*ble*Δ*polB*::ΩSpc *mutL211*::Tn*5*
RW718^a^	*recA730 lexA51*(Def) Δ(*umuDC*)*596*::*ermGT*Δ*dinB61*::*ble*Δ*polB*::ΩSpc *mutL211*::Tn*5*

a *thr-1 araD139*Δ(*gpt-proA*)*62 lacY1 tsx-33 glnV44 galK2 hisG4 rpsL31 xyl-5 mtl-1 argE3 thi-1 sulA211.*

b *sulA211 thi-1*Δ(*lac-gpt*)*5 ilv*(*Ts*) *mtl-1 rpsL31 supD43*.

c pCJ102 = F′ 5′→3′ exonuclease of pol I, Cm^R^ (Joyce and Grindley, 1984).

**Table 2:** Mutation rate for missense mutations in the *rpoB* gene of various *E. coli* strains.

Strain designation	Relevant genotype/phenotype	Mutation rate^a^ (×10^−8^)	Confidence limits (95%) (×10^−8^)
RW118	*recA*^+^*lexA*^+^*mutL*^+^	0.08	0.07–0.11
RW720	*recA*^+^*lexA*^+^*mutL*	16.7	14.9–18.6
RW740	*recA*^+^*lexA*^+^*dnaE486 mutL*	34.2	26.3–41.7
RW766	*recA*^+^*lexA*^+^*mutL*/pCJ102	6.2	4.7–8.3
RW742	*recA*^+^*lexA*^+^Δ*polA mutL*/pCJ102	3.8	3.4–5.4
RW722	*recA*^+^*lexA51*(Def) *mutL*	11.0	9.8–12.7
RW690	*recA730 lexA51*(Def) *mutL*	11.5	9.8–16.9
RW694	*recA730 lexA51*(Def) Δ*umuDC mutL*	10.9	8.3–13.2
RW708	*recA730 lexA51*(Def) Δ*dinB mutL*	13.6	11.2–15.2
RW712	*recA730 lexA51*(Def) Δ*polB mutL*	12.7	11.4–18.4
RW710	*recA730 lexA51*(Def) Δ*umuDC*Δ*dinB mutL*	9.8	8.3–13.7
RW714	*recA730 lexA51*(Def) Δ*umuDC*Δ*polB mutL*	17.4	13.8–20.6
RW716	*recA730 lexA51*(Def) Δ*dinB*Δ*polB mutL*	8.8	8.1–11.5
RW718	*recA730 lexA51*(Def) Δ*umuDC*Δ*dinB*Δ*polB mutL*	9.7	8.6–11.8
RW604	*recA730 lexA51*(Def) Δ*umuDC mutL*	8.5	6.5–9.7
RW604/pRW154	*recA730 lexA51*(Def) Δ*umuDC mutL* expressing UmuD′C	34.2	27.0–43.2
RW604/pRW144	*recA730 lexA51*(Def)Δ*umuDC mutL* expressing MucA′B)	45.0	37.3–50.3
RW604/pRW290	*recA730 lexA51*(Def) Δ*umuDC mutL* expressing RumA′B	46.3	24.3–72.0

a The *rpoB* mutation rates were calculated using the Jones method of the median (Jones *et al*., 1994) applied to 29–40 individual cultures, and by using the equation, μ = *m*/2*Nt* where μ is the mutation rate per generation, *m* is number of mutations per culture and *Nt* is the final number of cells in the culture (Armitage, 1952).

### Spectra of *rpoB* mutations in the presence of differential levels of *E. coli*'s five pols

**Fig. 1:**
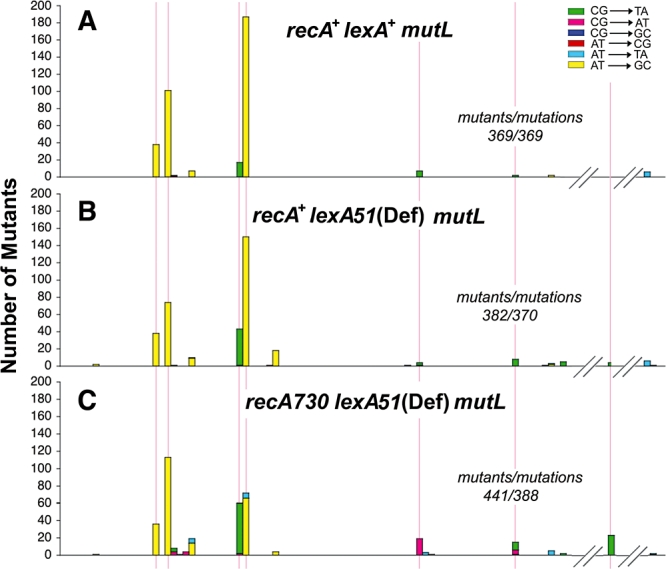
Spectrum of spontaneous *rpoB* mutations arising in various *mutL* strains. CG→TA transitions are coloured green; CG→AT transversion are in pink; CG→GC transversions are in dark blue; AT→CG transversions are in red; AT→TA transversions are in turquoise; and AT→GC transitions are in yellow. A. *recA*^+^*lexA*^+^*mutL* (RW720). B. *recA*^+^*lexA51*(Def) *mutL* (RW722). C. *recA730 lexA51*(Def) *mutL* (RW690).

The spectrum of spontaneous *rpoB* base-pair substitutions observed in a *recA*^+^*lexA*^+^*mutL* background is shown in Fig. 1A. As expected, transitions accounted for more than 90% of the total mutations observed (Table 3). Base-pair substitutions were located at 10 sites, with four prominent hot spots (defined as sites at which there are 10 or more mutations). These were AT→GC transitions located at positions 1532, 1534 and 1547 and a CG→TA transition at position 1546 (Table S1).

**Table 3:** Types of base-pair substitutions generated in *rpoB* in *recA*^+^*lexA*^+^*mutL211*::Tn5, *recA*^+^*lexA51*(Def) *mutL211*::Tn5 and *recA730 lexA51*(Def) *mutL211*::Tn5 strains.

Base-pair substitution	*recA*^+^*lexA*^+^*mutL*	*recA*^+^*lexA51*(Def) *mutL*	*recA730 lexA51*(Def) *mutL*
AT→TA	6	9	20
AT→CG	0	0	0
AT→GC	335	293	236
CG→GC	0	2	1
CG→AT	1	3	35
CG→TA	27	63	96

It is generally assumed that most genome duplication is performed by pol III holoenzyme with the help of pol I to seal Okazaki fragments (Kornberg and Baker, 1992). However, as noted in the introduction, both pol II and pol IV are expressed at reasonably high basal concentrations in an uninduced cell, so it is conceivable that both polymerases might contribute to spontaneous mutagenesis in wild-type cells. We were therefore interested in determining whether increasing the intracellular concentration of pols II and IV alters the spectrum of spontaneously arising *rpoB* mutations. To do so, we assayed *rpoB* mutagenesis in a *lexA*(Def) *mutL* strain (Fig. 1B), in which pols II and IV are expressed at maximally derepressed levels. Although both UmuD and UmuC are similarly expressed at high levels, the level of UmuD′ is very low, so the amount of active pol V under these conditions is negligible (Woodgate and Ennis, 1991). In general, the spectra of *rpoB* mutations in the *recA*^+^*lexA*(Def) *mutL* strain were very similar to the *recA*^+^*lexA*^+^*mutL* strain, with the exception that there was one additional AT→GC hot spot at position 1552.

Next, we determined the spectra of *rpoB* mutations in a *recA730 lexA51*(Def) *mutL* strain. In this background, all three LexA-regulated polymerases are fully expressed and the mutant RecA730 protein efficiently converts UmuD to UmuD′ in the absence of DNA damage (Shinagawa *et al*., 1988; Woodgate and Ennis, 1991), thereby activating pol V (Reuven *et al*., 1999; Tang *et al*., 1999). Interestingly, there was a noticeable difference in the *rpoB* spectra obtained in this background compared with either the *recA*^+^*lexA*^+^*mutL* or *recA*^+^*lexA*(Def) *mutL* backgrounds. In particular, there was a sharp drop in the number of AT→GC transition mutations at position 1547, as well as the appearance of multiple transversion mutations throughout the target region (Table 3), with the most notable being the CG→AT transversion at position 1576 (Table S1). As the transversion mutations were not observed in the *recA*^+^*lexA*(Def) *mutL* strain expressing pols I–IV, we hypothesize that the transversion events are most likely attributable to the mutagenic activity of pol V. Indeed, our observations are in good agreement with earlier studies showing that the pol V-dependent SOS mutator effect is characterized by a strong increase in transversion specificity (Yatagai *et al*., 1991; Miller and Low, 1984; Fijalkowska *et al*., 1997; Watanabe-Akanuma *et al*., 1997).

### Low-level expression of pol V is the limiting factor influencing transversion mutations

**Fig. 2:**
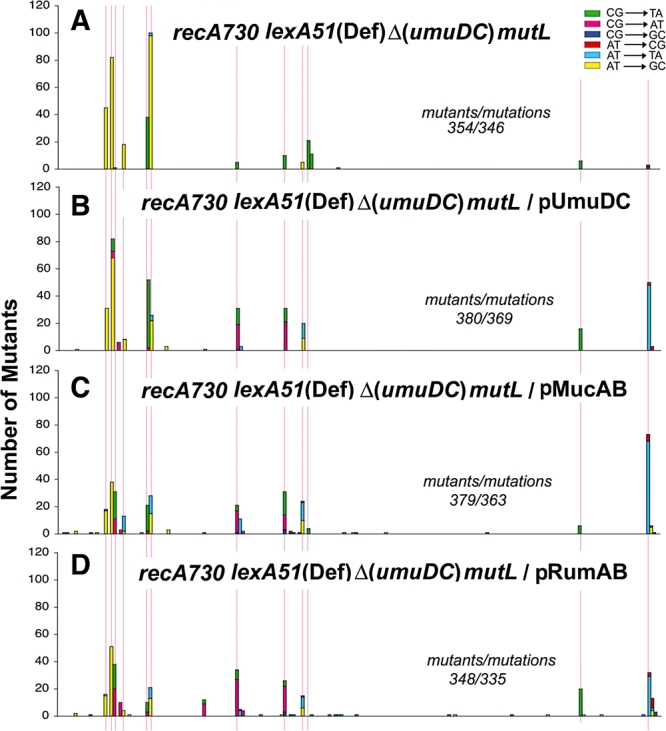
Spectrum of spontaneous *rpoB* mutations arising in *mutL* strains moderately overexpressing polV-like polymerases. CG→TA transitions are coloured green; CG→AT transversion are in pink; CG→GC transversions are in dark blue; AT→CG transversions are in red; AT→TA transversions are in turquoise; and AT→GC transitions are in yellow. A. *recA730 lexA51*(Def) *mutL* (RW604). B. *recA730 lexA51*(Def) *mutL*/pUmuDC (pRW154). C. *recA730 lexA51*(Def) *mutL*/pMucAB (pRW144). D. *recA730 lexA51*(Def) *mutL*/pRumAB (pRW290). Data for RW604 and RW604/pRW290 were taken from Mead *et al*. (2007) and are shown for comparison.

When maximally expressed in a *recA730 lexA*(Def) background, it is estimated that there are ∼700 molecules of pol V per cell (Woodgate and Ennis, 1991), largely because the RecA730 protein efficiently mediates conversion of UmuD to UmuD′, thereby circumventing both Lon- and ClpXP-degradation of UmuD (Frank *et al*., 1996). Under these conditions, levels of pol V are estimated to be approximately twofold higher than pol II (Bonner *et al*., 1988; Qiu and Goodman, 1997) and approximately three- to fourfold lower than pol IV (Kim *et al*., 2001). We were therefore interested in determining the effect of modest overproduction of pol V and the phylogenetically related pol R1 (MucA′B) and pol V_(R391)_ (RumA′B) on the spectrum of *rpoB* mutations in a Δ*umuDC lexA*(Def) *recA730 mutL* background. To this end, *E. coli* UmuDC, R46/pKM101 MucAB and R391 RumAB were expressed from the low-copy-number plasmid, pGB2 (Churchward *et al*., 1984). Interestingly, modest overproduction of the pol V-like enzymes in the *recA730 lexA*(Def) Δ*umuDC mutL* strain resulted in an ∼4- to 5.5-fold increase in the overall mutation rate compared with the *recA730 lexA*(Def) *mutL* strain (Table 2) and gave a very different distribution of spontaneous mutations, with the appearance of numerous hot spots and a substantial increase in the number of transversions (Fig. 2, Table 4, Table S2). Indeed, in the strain overexpressing *E. coli* pol V (UmuD′C), transversions accounted for ∼34% of the total base substitutions assayed. The spectrum was dominated by nine hot spots, among which CG→AT transversions were located at positions 1576, 1592 and AT→TA transversions at position 1714. Overexpression of pol R1 (R46/pKM101 MucA′B) also resulted in 32 additional mutation sites and a higher increase of the number of transversions, accounting for 51% of the total number of base substitutions. CG→AT transversions hot spots were recovered at positions 1535, 1576, 1592 and AT→TA transversions at positions 1538, 1547, 1577, 1598 and 1714. Similarly, overexpression of pol V_(R391)_ (R391 RumA′B) also caused a dramatic increase in the number of transversions, representing 10 of the 13 hot spots at positions 1535, 1537, 1546, 1565, 1576, 1577, 1592, 1598, 1714 and 1715. Comparison of the data presented in Fig. 1C with that of Fig. 2 therefore suggests that chromosomally encoded levels of pol V are the limiting factor for the production of spontaneous transversion mutations. This is evidenced by the fact that modest overproduction of pol V-like enzymes causes a dramatic increase in transversion hot spots, especially CG→AT mutations at positions 1535, 1576, 1598, 1691 and AT→TA mutations at positions 1598 and 1714. In general, the spectra of *rpoB* mutations generated in the presence of overproduced pol V-like enzymes was similar (Table S2), but there were also polymerase-specific hot spots, such as the pol R1-dependent AT→TA transversion at position 1538 and the pol V_(R391)_-dependent CG→AT transversion at position 1565.

**Table 4:** Types of base-pair substitutions generated in *rpoB* in a *recA730 lexA51* (Def) Δ*umuDC mutL211*::Tn5 strain in presence of UmuD′C, MucA′B and RumA′B.

Base-pair substitution	*recA730 lexA51*(Def) Δ(*umuDC*) *mutL*	*recA730 lexA51*(Def) Δ(*umuDC*) *mutL* pUmuDC	*recA730 lexA51*(Def) Δ(*umuDC*) *mutL* pMucAB	*recA730 lexA51*(Def) Δ(*umuDC*) *mutL* pRumAB
AT→TA	4	67	118	55
AT→CG	1	5	8	13
AT→GC	248	142	93	101
CG→GC	0	1	8	9
CG→AT	0	52	51	94
CG→TA	93	102	85	63

### Competition between *E. coli*'s polymerases in a *recA730 lexA*(Def) background

**Fig. 3:**
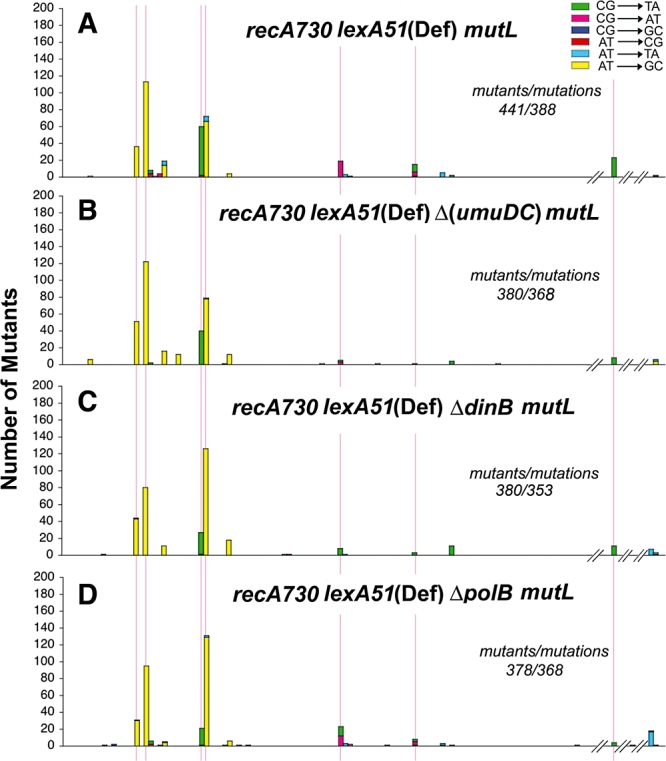
Spectrum of spontaneous *rpoB* mutations arising in *mutL* strains lacking pols II, IV or V. CG→TA transitions are coloured green; CG→AT transversion are in pink; CG→GC transversions are in dark blue; AT→CG transversions are in red; AT→TA transversions are in turquoise; and AT→GC transitions are in yellow. A. *recA730 lexA51*(Def) *mutL* (RW690). B. *recA730 lexA51*(Def) Δ*umuDC mutL* (RW694). C. *recA730 lexA51*(Def) Δ*dinB mutL* (RW708). D. *recA730 lexA51*(Def) Δ*polB mutL* (RW712). The data presented for RW690 are the identical to that reported in Fig. 1C and are shown for comparison.

The data presented in Figs 1 and 2 suggest that pol V is responsible for most of the transversion mutations recovered in *rpoB* in a *lexA*(Def) *recA730 mutL* background. To confirm this observation, we analysed the spectra of *rpoB* mutations in a Δ*umuDC lexA*(Def) *recA730 mutL* strain (Fig. 3B, Table 5, Table S3). As expected, in the absence of pol V, the number of AT→TA and CG→AT transversions dropped significantly. As controls for these experiments, we also determined the spectra of *rpoB* mutations recovered from a Δ*dinB lexA*(Def) *recA730 mutL* and Δ*polB lexA*(Def) *recA730 mutL* strain (Fig. 3C and D). Interestingly, in the Δ*dinB lexA*(Def) *recA730 mutL* strain there was an increase in AT→GC transitions at position 1547, as well as a dramatic decrease in the overall number of CG→AT transversions (Table 5). The latter suggests that in addition to pol V, pol IV is also involved in the generation of transversion mutations. Such observations are in agreement with Kuban *et al.* who reported that both pol IV and pol V are required to produce lagging-strand transversions in a *lacZ* reversion assay (Kuban *et al*., 2006). The spectrum of *rpoB* mutations recovered from the Δ*polB lexA*(Def) *recA730 mutL* strain was very similar to the isogenic *polB*^+^ strain. The major exceptions were a reduction in the GC→TA transition hot spot at position 1546 and a concomitant increase in the AT→GC transition hot spot at position 1547, and an increase in AT→TA transversions at position 1714.

**Table 5:** Types of base-pair substitutions generated in *rpoB* in *recA730 lexA*(Def) *mutL211*::Tn*5* strains lacking pol II, pol IV or pol V alone, or in combination.

Base-pair substitution	*recA730*^a^*lexA*(Def) *mutL*	*recA730lexA*(Def) Δ*umuDCmutL*	*recA730lexA*(Def) Δ*dinB mutL*	*recA730lexA*(Def) Δ*polB mutL*	*recA730lexA*(Def) Δ*dinB* Δ*umuDC mutL*	*recA730lexA*(Def) Δ*polB* Δ*umuDC mutL*	*recA730lexA*(Def) Δ*dinB* Δ*polB mutL*	*recA730lexA*(Def) Δ*dinB* Δ*polB* Δ*umuDC mutL*
AT→TA	20	4	11	26	5	12	8	11
AT→CG	0	0	2	5	0	3	0	0
AT→GC	236	303	279	265	323	285	349	270
CG→GC	1	0	0	3	0	0	0	0
CG→AT	35	3	2	24	0	7	3	2
CG→TA	96	58	59	45	43	56	18	79

a Data taken from Table 3 and shown for comparison.

**Fig. 4:**
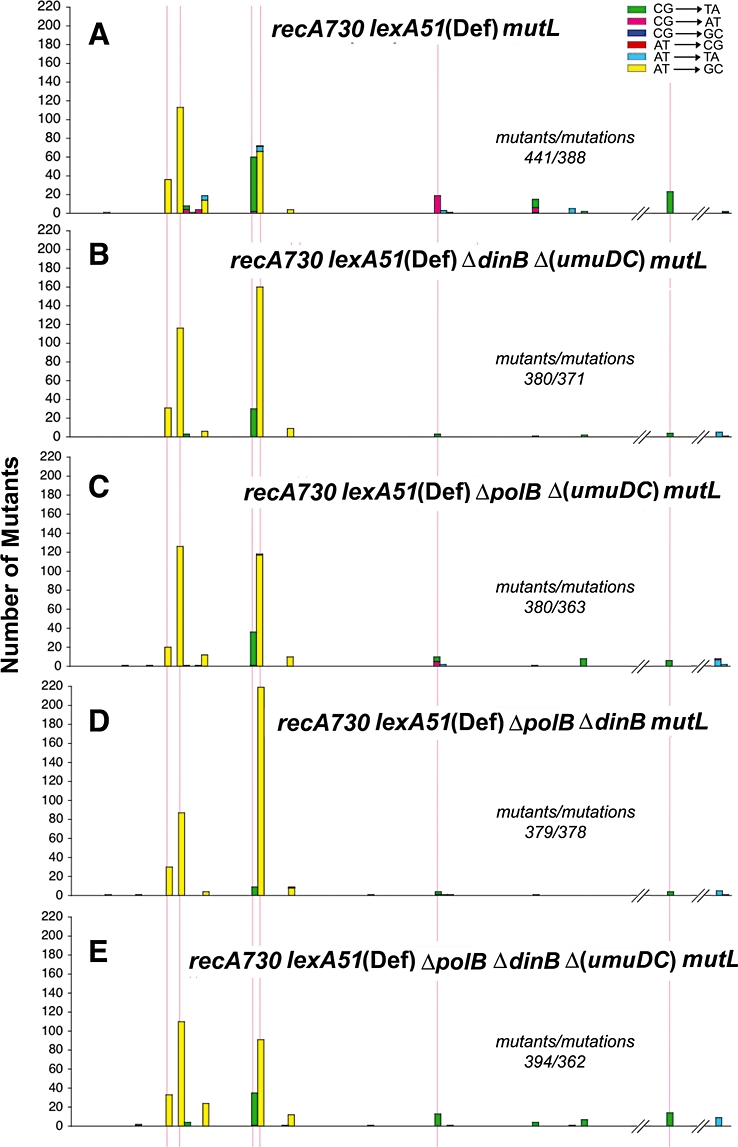
Spectrum of spontaneous *rpoB* mutations arising in *mutL* strains lacking a combination of pols II, IV or V. CG→TA transitions are coloured green; CG→AT transversion are in pink; CG→GC transversions are in dark blue; AT→CG transversions are in red; AT→TA transversions are in turquoise; and AT→GC transitions are in yellow. A. *recA730 lexA51*(Def) *mutL* (RW690). B. *recA730 lexA51*(Def) Δ*umuDC*Δ*dinB mutL* (RW710). C. *recA730 lexA51*(Def) Δ*umuDC*Δ*polB mutL* (RW714). D. *recA730 lexA51*(Def) Δ*polB*Δ*dinB mutL* (RW716). E. *recA730 lexA51*(Def) Δ*polB*Δ*dinB*Δ*umuDC mutL* (RW718). The data presented for RW690 are the identical to that reported in Fig. 1C and are shown for comparison.

Our observation that both pol IV and pol V may function together to promote transversions, and that pol II may also influence spontaneous mutagenesis at certain sites prompted us to analyse the spectra of *rpoB* mutations generated in a *recA730 lexA*(Def) *mutL* background in the absence of pols II, IV and V in various combinations (Fig. 4, Table 5 and Table S3). Indeed, in the absence of pol IV and pol V all of the CG→AT transversions disappeared, and only five AT→TA transversions were recovered at position 1714, thereby confirming that pol IV and V work in a common pathway to promote transversion mutations.

The *recA730 lexA*(Def) Δ*polB*Δ*umuDC mutL* strain exhibited the same general spectrum as the *recA730 lexA*(Def) Δ*umuDC mutL* strain alone (cf. Fig. 3B versus Fig. 4C). Similarly, the *recA730 lexA*(Def) Δ*polB*Δ*dinB mutL* double mutant gave a spectrum of *rpoB* mutations that was similar to the strain carrying a deletion of *dinB* alone (cf. Fig. 3C versus Fig. 4D), but which was dominated by an increase in the number of AT→GC transition mutations at position 1547. Thus, it appears that pols II and IV act to suppress transition mutations at this location. Interestingly, in the *recA730 lexA*(Def) *mutL* strain lacking pols II, IV and V, the number of AT→GC transition mutations at position 1547 drops considerably (Fig. 4E). As a significant number of AT→GC transitions at position 1547 were observed in the *recA730 lexA*(Def) Δ*umuDC mutL* strain (Fig. 4B), they are clearly not dependent upon pol V, but rather appear to be modulated by its presence. Last, but not least, the spectrum of mutations recovered from the *recA730 lexA*(Def) Δ*polB*, Δ*dinB*, Δ*umuDC mutL* strain (Fig. 4E) must reflect errors generated by either pol I or pol III, as they are the only known polymerases remaining in the triple deletion strain. The distribution of mutations was dominated by AT→GC transition hot spots at positions 1532, 1534, 1538, 1547 and 1552 and CG→TA transitions at positions 1546, 1576 and 1671. This spectrum largely resembled that observed in a *recA*^+^*lexA*(Def) *mutL* background (cf. Fig. 1B versus Fig. 4E). From this, we can infer that pols II, IV or V do not promote *rpoB* mutagenesis in a *recA*^+^*lexA*(Def) *mutL* strain and as a consequence, most spontaneous *rpoB* mutagenesis observed in a *recA*^+^*lexA*(Def) *mutL* strain is dependent upon pols I and III.

### Role of pol I and pol III in spontaneous mutagenesis

The data presented in Fig. 4E indicate that in a *recA730 lexA*(Def) *mutL* background, pol I or pol III is error-prone at particular hot spots within *rpoB*. To try and determine the relative contribution of each polymerase to spontaneous mutagenesis, we considered making *recA730 lexA*(Def) *mutL* strains with mutations in either *polA* or *dnaE* (encoding the catalytic α-subunit of pol III holoenzyme). However, *polA recA* mutants are inviable (Gross *et al*., 1971; Fijalkowska *et al*., 1989; Witkin and Roegner-Maniscalco, 1992) and *dnaE recA730 mutL* strains have very low viability (Fijalkowska *et al*., 1997). As a consequence, we analysed the effects of *polA* and *dnaE* in a *recA*^+^*lexA*^+^*mutL* background, where we have deduced that the four major hot spots in *rpoB* are largely attributed to misincorporation by pol I and/or pol III (cf. Fig. 1A versus Fig. 4E).

To analyse the role of pol III on *rpoB* mutagenesis we used *dnaE486*. This is a temperature-sensitive missense allele of *dnaE* caused by an S885P mutation (Vandewiele *et al*., 2002). This residue is not located close to the catalytic active site of the α-subunit, but presumably affects the ability of the α-subunit to interact with other subunits of the holoenzyme complex (Lamers *et al*., 2006). While *dnaE486* strains exhibit temperature-sensitive growth and are inviable at 43°C, they are able to grow at 37°C and at this temperature they exhibit a moderate mutator phenotype (Wechsler and Gross, 1971; Fijalkowska *et al*., 1997). This mutator phenotype is largely dependent upon pol V (Vandewiele *et al*., 2002), suggesting that pol V has greater access to genomic DNA in the *dnaE486* background.

**Fig. 5:**
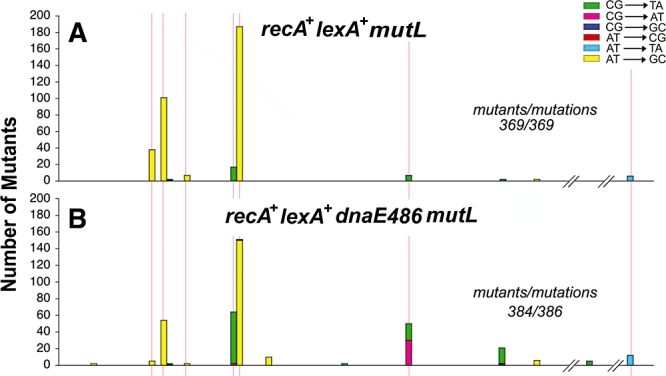
Effect of *dnaE486* on the spectrum of spontaneous *rpoB* mutations arising in a *recA*^+^*lexA*^+^*mutL* strain. CG→TA transitions are coloured green; CG→AT transversion are in pink; CG→GC transversions are in dark blue; AT→CG transversions are in red; AT→TA transversions are in turquoise; and AT→GC transitions are in yellow. A. *recA*^+^*lexA*^+^*mutL* (RW720). B. *recA*^+^*lexA*^+^*dnaE486 mutL* (RW740). The data presented for strain RW720 are identical to that reported in Fig. 1A and are shown for comparison.

Interestingly, the *recA*^+^*lexA*^+^*dnaE486 mutL* spectrum was very different to that of the *recA*^+^*lexA*^+^*mutL* control (Fig. 5, Table 6, Table S4). There was a significant decrease in AT→GC transitions at positions 1532 and 1534, suggesting that these hot spots could be pol III-dependent. Conversely, there was an increase in transversion mutations when compared with the isogenic *dnaE*^+^ strain (Table 6). In particular, we observed an increase in CG→TA transversions at position 1576, which we previously demonstrated are dependent upon pol IV and pol V (Figs 2A, B and 3B, Table S4).

**Table 6:** Types of base-pair substitutions generated in *rpoB* in *recA*^+^*lexA*^+^*dnaE*^+^*mutL211*::Tn*5* and *recA*^+^*lexA*^+^*dnaE486 mutL211*::Tn*5* strains.

Base-pair substitution	*recA*^+^*lexA*^+^*dnaE*^+^*mutL*^a^	*recA*^+^*lexA*^+^*dnaE486 mutL*
AT→TA	6	13
AT→CG	0	0
AT→GC	335	229
CG→GC	0	1
CG→AT	1	34
CG→TA	27	109

a Data taken from Table 3 and shown for comparison.

**Fig. 6:**
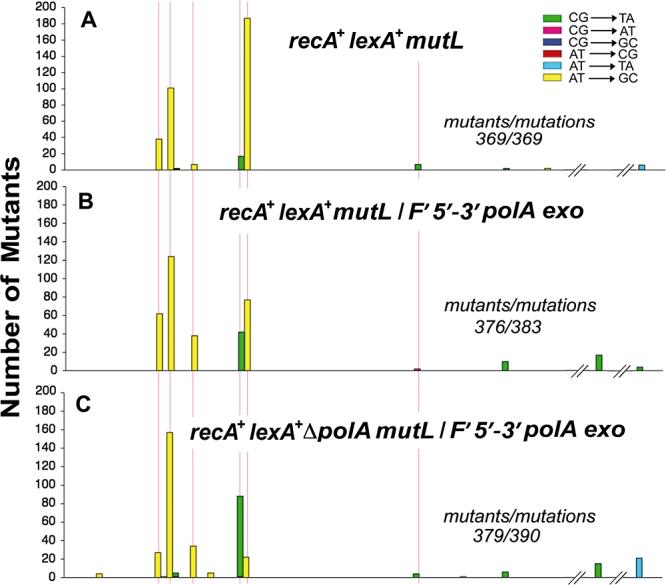
Effect of deleting *polA* on the spectrum of spontaneous *rpoB* mutations arising in *recA*^+^*lexA*^+^*mutL* strains. CG→TA transitions are coloured green; CG→AT transversion are in pink; CG→GC transversions are in dark blue; AT→CG transversions are in red; AT→TA transversions are in turquoise; and AT→GC transitions are in yellow. A. *recA*^+^*lexA*^+^*mutL* (RW720). B. *recA*^+^*lexA*^+^*mutL/*pCJ102 (F′ 5′-3′ pol I exonuclease) (RW766). C. *recA*^+^*lexA*^+^Δ*polA mutL*/pCJ102 (F′ 5′-3′ pol I exonuclease) (RW742). The data presented for strain RW720 are identical to that reported in Fig. 1A and are shown for comparison.

Δ*polA* strains are inviable when grown in rich media unless the strain carries a plasmid either expressing the 5′→3′ or 3′→5′ exonuclease domain of the polymerase (Joyce and Grindley, 1984). In our case, we used a strain carrying pCJ102, which expresses the 5′→3′ exonuclease of pol I (Joyce and Grindley, 1984). We first introduced the plasmid into the *recA*^+^*lexA*^+^*mutL* strain and assayed the spectrum of mutations in *rpoB* (Fig. 6, Table 7, Table S5). Somewhat surprisingly, we observed a significant decrease in the number of AT→GC transitions at position 1547 compared with the isogenic strain lacking pCJ102, as well as slight increases in the number of transitions at positions 1534 and 1546. Interestingly, deletion of pol I added to these effects in that there was a further decrease in the number of AT→GC transitions recovered at position 1547, and a concomitant increase in AT→GC and CG→TA transitions at positions 1534 and 1546 respectively. Thus, three of the four major transition hot spots within *rpoB* appear to be modulated by pol I, but in opposite ways. Pol I appears to suppress transition mutations arising at positions 1534 and 1546, but is largely responsible for the AT→GC transition mutation occurring at position 1547. These mutations presumably occur when pol I seals Okazaki fragments during genome duplication, or during the postreplicative gap-filling step of nucleotide excision repair. As noted above (Fig. 4), AT→GC transitions mutations at position 1547 increase significantly in the absence of pols II and IV. Thus, it appears that at this particular site, pols II and IV work together to suppress pol I-dependent misincorporation events.

**Table 7:** Types of base-pair substitutions generated in *rpoB* in *recA*^+^*lexA*^+^*mutL211*::Tn*5*, *recA*^+^*lexA*^+^*mutL218*::Tn*10*/*pCJ102* and *recA*^+^*lexA*^+^Δ*polA mutL218*::Tn*10*/pCJ102.

Base-pair substitution	*recA*^+^*lexA*^+^*mutL*^a^	*recA*^+^*lexA*^+^*mutL* pCJ102^b^	*recA*^+^*lexA*^+^ Δ*polA mutL* pCJ102
AT→TA	6	0	21
AT→CG	0	0	0
AT→GC	335	301	250
CG→GC	0	0	0
CG→AT	1	2	0
CG→TA	27	73	117

a Data taken from Table 3 and shown for comparison.

b pCJ102 = F′ 5′→3′ exonuclease of pol I, Cm^R^ (Joyce and Grindley, 1984).

## Discussion

The multiplicity of pols in *E. coli* requires that access of each polymerase to chromosomal DNA must be exquisitely regulated. To gain insights into the interplay that takes place among *E. coli*'s five pols, we have exploited the fact that each polymerase leaves a distinct ‘genetic fingerprint’ when copying DNA (Wolff *et al*., 2004). We analysed the spectrum of *rpoB* mutants generated in a set of 13 isogenic *mutL* strains. The use of strains defective in mismatch repair was imperative, as it allowed us to follow polymerase-specific misincorporation events, rather than assay mutations that escape repair. The strains also carried mutations in *recA* and *lexA* that lead to the differential expression of *E. coli*'s three SOS-regulated pols, pols II, IV and V. The *recA*^+^*lexA*^+^*mutL* strain served as the baseline for spontaneous mutagenesis in the absence of any SOS induction. In contrast, the *recA*^+^*lexA*(Def) *mutL* strain allowed us to determine the effects of pols II and IV when expressed at fully derepressed levels, and the *recA730 lexA*(Def) *mutL* strain, in which pol V is maximally activated, allowed us to investigate the potential competition between all five of *E. coli*'s pols. Furthermore, although we used genetically modified strains to derepress expression of pols II, IV and V, we believe that our observations can be extrapolated to wild-type *E. coli* cells growing under stressful conditions, where there is likely to be transient upregulation of pols II, IV and V.

Given that it is well known that mismatch repair largely protects an organism against transition mutations and has little effect on transversions (Schaaper and Dunn, 1987; 1993), it was no surprise that in our *rpoB* assays, the spectrum of mutations observed in the *recA*^+^*lexA*^+^*mutL* strain was dominated by AT→GC and CG→TA transitions at four well-defined hot spots (1532, 1534, 1546 and 1547). There was little difference in the spectrum of *rpoB* mutations recovered from the *recA*^+^*lexA*^+^*mutL* and *recA*^+^*lexA*(Def) *mutL* strains, suggesting that even when fully derepressed, pols II and IV are unable to compete with pol I and/or pol III for access to a nascent chromosomal primer. In contrast, in the *recA730 lexA*(Def) *mutL* strain, there was a dramatic change in the mutagenic spectrum with a shift from transition to transversion mutations. Thus, when fully derepressed, and maximally activated, it appears that pols II, IV and V are able to compete with pols I and III for access to genomic DNA and in doing so, either suppress or promote mutagenesis on the *E. coli* chromosome. For example, one of the most notable mutagenic events observed in *rpoB* was a dramatic increase in transitions at position 1547 in the *recA730 lexA*(Def) Δ*polB*Δ*dinB mutL* strain. By comparing *rpoB* mutagenesis in a *recA*^+^*lexA*^+^*mutL* strain to an isogenic strain carrying a deletion of *polA*, we determined that pol I is, in fact, responsible for most transition mutagenesis at position 1547. Such observations are remarkable given that pol I possesses both 3′→5′ and 5′→3′ exonuclease activities and is generally thought to be an accurate polymerase, with misincorporations occurring with a frequency of < 1 × 10^−6^ (Bebenek *et al*., 1990). The fact that mutagenesis at position 1547 increases in a Δ*polB* or Δ*dinB* strain is consistent with a potential ‘antimutator’ proofreading role by pol II (Banach-Orlowska *et al*., 2005; Gawel *et al*., 2008). However, pol IV lacks intrinsic exonuclease activity and presumably acts as a simple competitive inhibitor to prevent access to a primer terminus by error-prone pol I.

In contrast to the transition mutations, our study revealed that transversion mutations in a *recA730 lexA*(Def) *mutL* strain are largely dependent upon the combined actions of pol IV and pol V and that cellular levels of pol V are limiting for transversion mutagenesis.

The initial goal when embarking upon this study was that we would identify which particular DNA polymerase is responsible for specific mutagenic events within *rpoB*. Indeed, the AT→GC transition mutations observed at position 1547 and the CG→AT transversions at position 1576 are generated through the mutagenic actions of pol I and pol IV/V respectively. In contrast, pol II appears to play a largely antimutagenic role by suppressing mutagenesis at certain hot spots. Until now, it has tacitly been assumed that spontaneous mutations occurring in the *E. coli* genome largely arise during genome duplication performed by the cell's replicase, pol III. This may be true for the transition mutations recovered at positions 1534 and 1546. However, based upon our observations above, we also have to conclude that under certain conditions, there is considerable switching among *E. coli*'s five pols and that such interplay modulates the extent of spontaneous mutagenesis occurring on the *E. coli* chromosome, especially in cells growing under stressful conditions.

## Experimental procedures

### Bacterial strains and plasmids

Most of the *E. coli* K-12 strains used in this study are derivatives of RW118 (full genotype: *thr-1 araD139*Δ(*gpt-proA*)*62 lacY1 tsx-33 supE44 galK2 hisG4 rpsL31 xyl-5 mtl-1 argE3 thi-1 sulA211*) (Ho *et al*., 1993). The exception was strain RW604 [full genotype: *thi-1*Δ(*lac-gpt*)*5 ilv*(*Ts*) *mtl-1 rpsL31 supD43 recA730 srlC300*::Tn*10 lexA51*(Def) *sulA211*Δ*umuDC595*::*cat mutL211*::Tn*5*] (Mead *et al*., 2007) (Table 1). All derivatives of RW118 were made by standard methods of P1 transduction using P1*vir* (Table 1). The various polymerase or *mutL* alleles used in this study were obtained from the following strains: CJ231 for Δ*polA*::kan (Joyce and Grindley, 1984); STL1366 for Δ*polB*::Ωspec (Rangarajan *et al*., 1999); AR30 for Δ*dinB61*::*ble* (Borden *et al*., 2002); EC8 for Δ*umuDC596*::*ermGT* (Frank *et al*., 1996); RW620 for *dnaE486 zae502*::Tn*10* (Rangarajan *et al*., 1999); NR10775 for *mutL211*::Tn*5* (Schaaper, 1996); and ES1484 for *mutL218*::Tn*10* (Siegel *et al*., 1982).

Low-copy-number plasmids derived from pGB2 (Churchward *et al*., 1984), which encode *E. coli umuDC* (pRW154), R46/pKM101 *mucAB* (pRW144) or R391 *rumAB* (pRW290), have been described previously (Ho *et al*., 1993; Szekeres *et al*., 1996; Mead *et al*., 2007).

Where noted, bacteria were grown on LB agar plates containing 20 μg ml^−1^ chloramphenicol, 15 μg ml^−1^ tetracycline, 25 μg ml^−1^ zeocin, 50 μg ml^−1^ kanamycin, 50 μg ml^−1^ spectinomycin and 100 μg ml^−1^ rifampicin.

### Colony PCR assay to test for *ΔpolA*, *ΔpolB*, *ΔdinB* and *ΔumuDC* genotypes

Although the Δ*polA*, Δ*polB*, Δ*dinB* and Δ*umuDC* substitution alleles are all marked with a selectable antibiotic resistance, we encountered situations where we obtained a large number of antibiotic-resistant colonies (especially with Δ*umuDC*::*ermGT*) that ultimately turned out to be false positives. As a consequence, all transductants were initially selected by growth on the appropriate antibiotic-containing media and subsequently confirmed by colony PCR.

The primers used to detect the Δ*polA*::kan allele were POLA1 (5′-TTC-CGA-CCA-TCA-AGC-ATT-TTA-T-3′) and POLA2 (5′-TCA-GGC-ATT-ACG-GAT-CTT-TTC-T-3′). The temperature profile used with these primers was 25 cycles at 94°C for 1 min, 50°C for 1 min and 72°C for 2 min, which results in the amplification a 923 bp PCR fragment.

The primers used to detect the Δ(*araD-polB*)::Ω allele were SPCR2 (5′-TCT GTC CTG GCT GGC GAA CGA-3′) and POLB (5′-CCG ACG GGA TCA ATC AGA AAG GTG-3′). The temperature profile used with these primers was 25 cycles at 94°C for 1 min, 55°C for 1 min and 72°C for 2 min, which results in the amplification an 817 bp PCR fragment.

The primers used to detect the Δ*dinB61*::*ble* allele were AR270 (5′-GCC ATG ACC GAG ATC GGC GAG CAG CC-3′) and AR271 (5′-TGT ATA CTT TAC CAG TGT TGA GAG G-3′). The temperature profile used with these primers was 35 cycles of 95°C for 30 s, 60°C for 1 min and 72°C for 1 min, which results in the amplification a 318 bp PCR fragment.

The primers used to detect the Δ(*umuDC*)*596*::*ermGT* allele were EMR1 (5′-GCG-GGA-TGC-GTC-AAT-GTC-3′) and ERM2 (5′-CAC-CCT-TCA-AAA-ATA-TCA-CTC-AAA-3′). The temperature profile used with these primers was 25 cycles at 94°C for 1 min, 50°C for 1 min and 72°C for 2 min, which results in the amplification a 1155 bp PCR fragment.

### Spectra of spontaneous mutations in *rpoB*

The mutation spectra were generated using the *rpoB*/Rif^R^ mutagenesis assay developed by Jeffery Miller's group (Garibyan *et al*., 2003; Wolff *et al*., 2004). The *rpoB* gene encodes the β-subunit of RNA polymerase and base-pair substitutions in *rpoB* are either lethal or result in rifampicin resistance. Eighty-eight per cent of all *rpoB* mutations are localized in the central 202 bp region of the gene (Garibyan *et al*., 2003), thus offering the advantage that a single pair of oligonucleotide primers can be used for PCR amplification, and a single primer for DNA sequencing (Mead *et al*., 2007). The protocol used to identify *rpoB* mutations in the various strain backgrounds is detailed below. Each strain was diluted from a frozen stock culture and plated on the appropriate antibiotic-containing Luria–Bertani (LB) agar plates in order to obtain at least 600 independent colonies. Plates were incubated at 37°C overnight. Single colonies were picked and used to inoculate ∼600 independent LB cultures per strain. Cultures were grown for 24 h at 37°C and streaked on an LB agar plate containing 100 μg ml^−1^ rifampicin. A single Rif^R^ colony was picked from each streak. To avoid bias based on colony size, the Rif^R^ colony closer to a predetermined target point was chosen from each streak. Colony PCR was then performed on individual colonies in a 96-well microtitre plate. An ∼1 kb central region of the *rpoB* gene was amplified using the PCR primers RpoB1: 5′-CAC ACG GCA TCT GGT TGA TAC AG-3′ and RpoF1: 5′-TGG CGA AAT GGC GGA AAA C-3′. Amplification was achieved by denaturation at 95°C for 3 min, followed by 30 cycles of 94°C for 30 s, 1 min at 59°C, 2 min at 72°C, followed by a final extension step at 72°C for 7 min. Primer WOG923AP01 (5′-CAG TTC CGC GTT GGC CTG-3′) was used to determine the nucleotide sequence of the 300 bp target region of *rpoB* in each PCR amplicon (Cogenics, Houston, TX). Only base-pair substitutions occurring between positions 1516 and 1717 of the *rpoB* gene were considered during data analysis. Nucleotide sequences obtained were aligned and analysed using the SeqManager program of the DNASTAR suite (Madison, WI).

For derivatives of RW604 harbouring pRW154, pRW144 or pRW290, the protocol was modified slightly. Instead of initially diluting cultures from a frozen stock culture, RW604 was freshly transformed with plasmid DNA and ∼600 individual transformants were immediately picked for subsequent *rpoB* analysis (Mead *et al*., 2007).

### Determination of mutation rates

Each strain was diluted from a frozen stock culture into fresh liquid media to give an initial count of ∼100 cells ml^−1^ and grown overnight at 37°C. Aliquots (50 μl) of each overnight culture were plated on agar plates containing 100 μg ml^−1^ of rifampicin for selection of the forward mutation in *rpoB*. Plates were incubated overnight at 37°C. The number of viable colonies in the culture was determined by simply plating 50 μl of the appropriate serial dilution (in PBS media) onto LB agar plates in duplicate, followed by overnight incubation at 37°C. For the wild-type *mutL*^+^ strain, RW118, 3 ml cultures were harvested by centrifugation and re-suspended in an equal volume of PBS. The *rpoB* mutation rates were calculated as recommended (Foster, 2006), using the Jones method of the median (Jones *et al*., 1994) applied to 29–40 individual cultures and using the equation, μ = *m*/2*Nt*, where μ is the mutation rate per generation, *m* is number of mutations per culture and *Nt* is the final number of cells in the culture (Armitage, 1952).

## Supporting information

Additional supporing information may be found in the online version of this article.
