# Supplementary material for: DNA polymerase switching: effects on spontaneous mutagenesis in Escherichia coli
Source: Mol Microbiol. 2008 Nov 20;71(2):315–31. doi: 10.1111/j.1365-2958.2008.06526.x (PMC2680738; doi:10.1111/j.1365-2958.2008.06526.x)
Supplement: Supplementary file 1 [file mmi0071-0315-SD1.pdf]

Supplementary Table 1. Spectrum of spontaneous mutations generated in *rpoB* in *recA*$^{+}$ *lexA*$^{+}$ *mutL211*::Tn*5*, *recA*$^{+}$ *lexA51*(Def) *mutL211*::Tn*5* and *recA730 lexA51*(Def) *mutL211*::Tn*5* strains

Position[a]	bp change	*recA*$^{+}$ *lexA*$^{+}$ *mutL*	*recA*$^{+}$ *lexA51*(Def) *mutL*	*recA730 lexA51*(Def) *mutL*	Position	bp change	*recA*$^{+}$ *lexA*$^{+}$ *mutL*	*recA*$^{+}$ *lexA51*(Def) *mutL*	*recA730 lexA51*(Def) *mutL*
1522	AT→GC		2	1	1576	CG→AT		1	19
1532	AT→GC	38	38	36	1576	CG→TA	7	3	
1534	AT→GC	101	74	113	1577	AT→TA			3
1535	CG→AT	1		4	1578	CG→AT			1
1535	CG→TA	1	1	4	1592	CG→AT			5
1536	AT→GC			1	1592	CG→GC			1
1537	CG→AT			4	1592	CG→TA	2	8	9
1538	AT→GC	7	9	14	1597	CG→GC		1	
1538	AT→TA		1	5	1598	AT→GC	2	2	
1546	CG→TA	17	42	58	1598	AT→TA		1	5
1546	CG→AT		1	2	1600	CG→TA		5	2
1547	AT→GC	187	150	66	1691	CG→TA		4	23
1547	AT→TA			6	1714	AT→TA	6	6	
1551	CG→AT		1		1715	AT→TA		1	1
1552	AT→CG		18	4	1715	AT→GC			1
1574	CG→GC		1		Total		369	370	388

[a]: The numbering system originates from Garibyan *et al*., (2003), where the A of the ATG initiation codon is #1.

Supplementary Table 2. Spectrum of spontaneous mutations generated in *rpoB* in a *recA730 lexA51*(Def) Δ*umuDC mutL211*::Tn*5* strain in the presence of UmuD'C, MucA'B or RumA'B

Position[a]	bp change	*recA730 lexA*(Def) *Δ(umuDC) mutL*[b]	*recA730 lexA*(Def) *Δ(umuDC) mutL* pUmuDC	*recA730 lexA*(Def) *Δ(umuDC) mutL* pMucAB	*recA730 lexA*(Def) *Δ(umuDC) mutL* pRumAB[b]
1518	CG→TA			1	
1519	CG→AT			1	
1522	AT→GC		1	2	2
1527	CG→AT			1	
1527	CG→GC				1
1529	AT→GC			1	
1532	AT→TA				1
1532	AT→CG			1	
1532	AT→GC	45	31	17	15
1534	AT→GC	82	68	38	51
1535	CG→AT		5	11	20
1535	CG→TA	1	9	20	18
1537	CG→AT		6	3	10
1538	AT→GC	18	8	2	4
1538	AT→TA			11	
1544	AT→GC			1	
1540	AT→GC				1
1546	CG→AT		2	2	3
1546	CG→TA	38	50	19	7
1547	AT→GC	98	22	15	13
1547	AT→TA	2	4	13	8
1552	AT→GC		3		
1565	AT→TA		1		
1565	CG→AT			1	9
1565	CG→TA				3
1576	CG→GC		1	1	
1576	CG→AT		18	16	27
1576	CG→TA	5	12	4	7
1577	AT→CG				1
1577	AT→TA		3	11	4
1578	CG→AT			2	4
1584	AT→TA				1
1591	AT→GC			2	1
1592	CG→AT		21	11	19
1592	CG→GC			3	3
1592	CG→TA	10	10	17	4

Position[a]	bp change	*recA730 lexA*(Def) *Δ(umuDC) mutL*[b]	*recA730 lexA*(Def) *Δ(umuDC) mutL* pUmuDC	*recA730 lexA*(Def) *Δ(umuDC) mutL* pMucAB	*recA730 lexA*(Def) *Δ(umuDC) mutL* pRumAB[b]
1594	CG→GC			2	1
1595	CG→TA				1
1595	CG→AT			1	
1597	CG→GC			1	
1598	AT→TA		11	13	8
1598	AT→GC	5	9	10	6
1598	AT→CG			1	1
1600	CG→TA	21	4	4	
1601	CG→TA	11	1		
1607	CG→TA				1
1609	CG→GC				1
1610	CG→TA	1			1
1611	AT→TA				1
1612	CG→TA			1	
1615	AT→GC			1	
1616	AT→CG			1	
1616	CG→AT				1
1626	AT→GC			1	
1642	CG→AT				1
1647	CG→TA				1
1649	AT→GC				1
1660	CG→AT			1	
1668	AT→CG				1
1680	AT→GC				1
1691	CG→TA	6	16	19	20
1692	AT→GC				1
1698	AT→GC				1
1712	AT→TA				1
1714	AT→CG	1	2	5	3
1714	AT→TA	2	48	68	29
1715	AT→TA				2
1715	AT→GC			5	4
1715	AT→CG		3	1	7
1716	CG→GC			1	3
Total		346	369	363	336

[a] : The numbering system originates from Garibyan *et al*., (2003), where the A of the ATG initiation codon is #1.
[b]: Data taken from Mead *et al*., (2007) and shown for comparison.

Supplementary Table 3. Spectrum of spontaneous mutations generated in *rpoB* in *recA730 lexA*(Def) *mutL211*::Tn*5* strains lacking pol II, pol IV or pol V alone, or in combination

Position[a]	bp changed	*recA730* [b] *lexA*(Def) *mutL*	*recA730 lexA*(Def) *ΔumuDC mutL*	*recA730 lexA*(Def) *ΔdinB mutL*	*recA730 lexA*(Def) *ΔpolB mutL*	*recA730 lexA*(Def) *ΔdinB ΔumuDC mutL*	*recA730 lexA*(Def) *ΔpolB ΔumuDC mutL*	*recA730 lexA*(Def) *ΔdinB ΔpolB mutL*	*recA730 lexA*(Def) *ΔdinB ΔpolB ΔumuDC mutL*
1522	AT→GC	1	6					1	
1525	AT→CG			1	1		1		
1527	CG→GC				2				
1527	CG→AT							1	1
1527	CG→TA								1
1529	AT→CG						1		
1532	AT→GC	36	51	43	30	31	20	30	33
1532	AT→TA				1				
1532	AT→CG			1					
1534	AT→GC	113	122	80	95	116	126	87	110
1535	CG→AT	4			2				
1535	CG→TA	4	2		4	3	1		4
1536	AT→GC	1							
1537	CG→AT	4			1		1		
1538	AT→GC	14	16	11	4	6	12	4	24
1538	AT→TA	5			1				
1541	AT→GC		12						
1546	CG→AT	2		1	1		1		1
1546	CG→TA	58	40	26	20	30	35	9	34
1547	AT→GC	66	78	126	129	160	117	219	91
1547	AT→TA	6	1		2		1		
1551	CG→TA		1		1			1	1
1552	AT→GC	4	12	18	6	9	10	8	12
1554	CG→AT				1				
1556	AT→CG				1				
1564	AT→TA			1					
1565	CG→AT			1				1	1
1572	AT→GC		1						
1576	CG→AT	19	3		12		5		
1576	AT→TA							1	
1576	CG→TA		2	8	11	3	5	3	13
1577	AT→TA	3		1	3		2	1	1

Supplementary Table 3 continued. Spectrum of spontaneous mutations generated in *rpoB* in *recA730 lexA51*(Def) *mutL211*::Tn*5* strains lacking pol II, pol IV or pol V alone, or in combination.

Position[a]	bp changed	*recA730* [b] *lexA*(Def) *mutL*	*recA730 lexA*(Def) *ΔumuDC mutL*	*recA730 lexA*(Def) *ΔdinB mutL*	*recA730 lexA*(Def) *ΔpolB mutL*	*recA730 lexA*(Def) *ΔdinB ΔumuDC mutL*	*recA730 lexA*(Def) *ΔpolB ΔumuDC mutL*	*recA730 lexA*(Def) *ΔdinB ΔpolB mutL*	*recA730 lexA*(Def) *ΔdinB ΔpolB ΔumuDC mutL*
1578	CG→AT	1			2			1	
1584	AT→TA		1						
1586	CG→AT				1				
1592	CG→GC	1			1				
1592	CG→AT	5			4				
1592	CG→TA	9	1	3	3	1	1	1	4
1598	AT→GC				1				
1598	AT→TA	5			2				1
1600	CG→TA	2	4	11	1	2	8		7
1623	CG→TA				1				
1626	AT→GC		1						
1691	CG→TA	23	8	11	4	4	6	4	14
1710	AT→CG				1				
1714	AT→CG	1			1		1		
1714	AT→TA	1		7	17	5	7	5	9
1715	AT→GC		4	1		1			
1715	AT→TA		2	2			2	1	
1715	AT→CG				1				
	Total	388	368	353	368	371	363	378	362

[a] The numbering system originates from Garibyan *et al*., (2003), where the A of the ATG initiation codon is #1.
[b]: Data taken from Supplementary Table 1 and shown for comparison.

Supplementary Table 4. Spectrum of spontaneous mutations generated in *rpoB* in *lexA*[+] *recA*[+] *dnaE*[+] *mutL211*::Tn*5 and recA lexA*[+ +] *dnaE486 mutL211*::Tn*5*

Position[a]	bp change	*recA*[+ b] *lexA*[+] *mutL*	*recA*[+] *lexA*[+] *dnaE486 mutL*	Position	bp change	*recA*[+ b] *lexA*[+] *mutL*	*recA*[+] *lexA*[+] *dnaE486 mutL*
1522	AT→GC		2	1547	AT→TA		1
1532	AT→GC	38	5	1552	AT→CG		10
1533	CG→TA			1565	CG→TA		2
1534	AT→GC	101	54	1576	CG→AT		30
1535	CG→AT	1	1	1576	CG→TA	7	20
1535	CG→TA	1	1	1584	AT→CG		
1536	AT→GC			1592	CG→AT		1
1537	CG→AT			1592	CG→GC		1
1538	AT→GC	7	2	1592	CG→TA	2	19
1541	AT→GC			1598	AT→GC	2	6
1546	CG→TA	17	62	1691	CG→TA		5
1546	CG→AT		2	1714	AT→TA	6	12
1547	AT→GC	187	150	Total		369	386

[a]: The numbering system originates from Garibyan *et al.*, (2003), where the A of the ATG initiation codon is #1.
[b]: Data taken from Supplementary Table 1 and shown for comparison.

Supplementary Table 5. Spectrum of spontaneous mutations generated in *recA*$^{+}$ *lexA*$^{+}$ *mutL211*::Tn*5*, *recA*$^{+}$ *lexA*$^{+}$ *mutL218*::Tn*10* /pCJ102 and *recA*$^{+}$ *lexA*$^{+}$ Δ*polA* *mutL218*::Tn*10* /pCJ102 strains

Position[a]	bp change	*recA*$^{+}$[b] *lexA*$^{+}$ *mutL*	*recA*$^{+}$ *lexA*$^{+}$ *mutL* pCJ102[c]	*recA*$^{+}$ *lexA*$^{+}$ *ΔpolA* *mutL* pCJ102	Position	bp change	*lexA*$^{+}$[b] *recA*$^{+}$	*recA*$^{+}$ *lexA*$^{+}$ *mutL* pCJ102	*recA*$^{+}$ *lexA*$^{+}$ *ΔpolA* *mutL* pCJ102
1522	AT→GC			4	1547	AT→TA			
1532	AT→GC	38	32	27	1552	AT→CG			
1533	CG→TA			1	1565	CG→TA			
1534	AT→GC	101	124	157	1576	CG→AT			
1535	CG→AT	1		1	1576	CG→TA	7		4
1535	CG→TA	1		4	1584	AT→CG			1
1536	AT→GC				1592	CG→AT			
1537	CG→AT				1592	CG→GC			
1538	AT→GC	7	38	34	1592	CG→TA	2	2	6
1541	AT→GC			5	1598	AT→GC	2		
1546	CG→TA	17	42	87	1691	CG→TA		17	15
1546	CG→AT			1	1714	AT→TA	6	4	21
1547	AT→GC	187	77	22	Total		369	376	390

[a]: The numbering system originates from Garibyan *et al*., (2003), where the A of the ATG initiation codon is #1.
[b]: Data taken from Supplementary Table 1 and shown for comparison.
[C]: pCJ102 = F' 5'-3' polA exonuclease
